# Special Agents Can Promote Cooperation in the Population

**DOI:** 10.1371/journal.pone.0029182

**Published:** 2011-12-21

**Authors:** Xin Wang, Jing Han, Huawei Han

**Affiliations:** Key Laboratory of Systems and Control, Institute of Systems Science, Academy of Mathematics and Systems Science, Chinese Academy of Sciences, Beijing, China; Hungarian Academy of Sciences, Hungary

## Abstract

Cooperation is ubiquitous in our real life but everyone would like to maximize her own profits. How does cooperation occur in the group of self-interested agents without centralized control? Furthermore, in a hostile scenario, for example, cooperation is unlikely to emerge. Is there any mechanism to promote cooperation if populations are given and play rules are not allowed to change? In this paper, numerical experiments show that complete population interaction is unfriendly to cooperation in the finite but end-unknown Repeated Prisoner's Dilemma (RPD). Then a mechanism called soft control is proposed to promote cooperation. According to the basic idea of soft control, a number of special agents are introduced to intervene in the evolution of cooperation. They comply with play rules in the original group so that they are always treated as normal agents. For our purpose, these special agents have their own strategies and share knowledge. The capability of the mechanism is studied under different settings. We find that soft control can promote cooperation and is robust to noise. Meanwhile simulation results demonstrate the applicability of the mechanism in other scenarios. Besides, the analytical proof also illustrates the effectiveness of soft control and validates simulation results. As a way of intervention in collective behaviors, soft control provides a possible direction for the study of reciprocal behaviors.

## Introduction

Since Darwin's evolutionary theory, researchers have been long puzzled by the problem that why there exists wide cooperation among species [1]–[3]. As the paradigm of studying reciprocal behaviors, the Prisoner's Dilemma has been abstracted to depict many biological processes [4]–[8], and it raises a question to us, how to sustain cooperation in the group of self-interested agents without centralized control.

As known, in a single shot of two-agent Prisoner's Dilemma, mutual defection is the only equilibrium. With the number of agents increasing, it becomes unfriendly to cooperation either [9]. A large amount of theoretical work have studied assorted scenarios where cooperation can emerge. They can be mainly divided into three categories. First, the “catalysis” to sustain cooperation is studied. A specific proportion of “Tit for Tat” (TFT) in the population is crucial to the emergence of cooperation but the strategy of “Pavlov” is the last laughter [10], [11]. Punishment is considered as an important way to support cooperative behaviors and studied in spatial public goods game [12], [13], indirect reciprocity [14]–[16], group selection [17], [18] or other scenarios [19]–[22]. Besides costly punishment, reward can also promote cooperation [23]. Second, extra abilities or characteristics are provided to agents. The tag mechanism where an agent's decision depends not only on its play strategy but also on arbitrary tags associated with the agents can make it easy for populations to reach reciprocal cooperation [24], [25]. The mobility of an agent who interacts with its local neighbors also increases the capability of cooperation to emerge [26]–[29]. Third, introducing the topological structure in games, e.g. the lattice, the random graph or the scale-free network, has been proved to be an effective way to support cooperation because local interaction provides an opportunity for cooperators to cluster, grow and resist against the invasion by defectors [30]–[38]. Different samplings of interaction partners have effect on the cooperation level [39]. In addition, the introduction of coevolutionary rules combining the evolution of play strategies and other properties is beneficial to the prevalence of cooperation [40]–[49].

In this paper, our purpose is not to study which scenario can favor cooperation, but to propose a mechanism called soft control [50], [51] to promote cooperation in the unfavorable scenario. Moreover sometimes original populations and play rules are not allowed to alter because any change may incur high cost. Thus it is natural to ask how to promote cooperation under this circumstance. According to the basic idea of soft control, a number of special agents called shills are added to the original group to intervene in the evolution of cooperation. These shills pose as normal agents by conforming to play rules, thus they are always treated as normal agents by truly normal ones. The difference is that a shill has its own strategy and it can recognize other shills. This allows shills to share their knowledge of interacting with normal agents and take appropriate action in games based on knowledge. We think that this assumption is reasonable in some scenario of real life. Consider e-commerce: in order to publicize products, some sellers may employ a number of shills to compliment products in web media. Those shills recognize each other if the seller informs them, but ordinary consumers cannot differentiate. The preliminary result of soft control to promote cooperation in the particular scenario can be found in [52].

In following parts we study the performance of soft control under different settings by numerical experiments, which include: (1) the short-term vs. long-term RPD; (2) noise-free vs. noisy interaction; (3) complete vs. incomplete population interaction. For (1), our purpose is to show how the mechanism takes effect upon a wide range of the finitely RPD. Here note that albeit the finitely RPD is considered in this paper, we always assume that the time period of games is unknown to all players (i.e. normal agents and shills). So under this circumstance the finitely RPD is usually considered as the infinitely RPD. There are many theoretical studies based on the finitely RPD [1], [53]–[56]. For (2), the sensitivity of soft control to noise is presented where noise derives from mistakes to take the opposite action. The motivation of this experiment is to check whether the mechanism is robust to noise because any single-bit error in action between two TFT agents will destroy cooperation. It is called “cascade of curse”. For (3), we derive our main results in this paper on considering complete population interaction, i.e. every player plays with all others. A case of incomplete interaction is studied to demonstrate the applicability of soft control in other scenarios. In addition, we also give the analytical proof of the effectiveness under complete interaction (see in Appendix S1) to validate and complement simulation results.

## Methods

In the Prisoner's Dilemma, both players make their choices simultaneously, cooperation (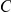) or defection (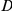). Their payoffs depend on which action they choose. The payoff matrix considered in this paper is written as the following form:
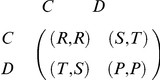
where parameters in the matrix satisfy 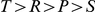 and 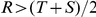.

According to the above matrix, note that in a single stage of the game 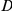 is the best option for a player regardless of its opponent's choice. As a result both obtain 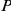 points. But if they had cooperated with each other, they would have received higher payoffs, 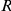 points. This is the dilemma between individual and collective rationality. Meanwhile playing 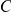 continuously is better than doing 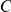 and 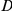 alternatively for the study of reciprocal behaviors in the RPD.

### Basic model: populations and play rules

Consider the mixed reactive strategy [57] for each normal agent, which is described as 
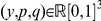
, where 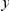 is the probability of cooperating on the first stage, 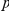 and 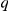 are the conditional probability of taking cooperation on the current stage responding to the opponent's last move defection and cooperation respectively. The space of reactive strategies displays plentiful phenomena and has rich analytical results [10], [24], [54], [57]–[59]. It can also describe the fundamental element in decision makings, i.e. the mapping from stimulus to response, similar to if-then rule.

Let 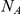 denote as the number of normal agents, 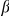 as the time period of games (e.g. 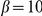 means the 10-stage RPD) and 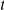 as the index of generations. Let 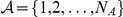. We assume the number of the population to be constant in each generation.

This paper mainly studies complete population interaction, i.e. each normal agent interacts with all others (in simulations, the incomplete interaction cases are also provided). In 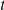 generation (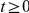), every pair of agents play the 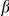-stage RPD once. The pairing order is random, but it does not influence an agent's payoff. Agent 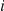 (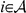) updates its payoff after each RPD game. Let 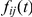 denote as the total payoff agent 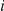 receives from playing with agent 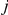 for the 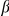-stage RPD. Then agent 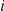
's total payoff 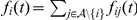. At the end of a generation, all agents self-reproduce. The expected number of agent 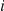
's offspring in 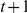 generation, denoted as 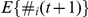, is calculated as follows:
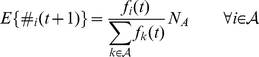
(1)As we will show in the simulation section, cooperation is impossible to emerge in the self-organized group of normal agents.

### Model with soft control: shills and their strategies

To promote cooperation, a number of shills are added to the original group. Let 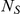 denote as the number of shills. Again, we assume the number of the population 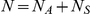 to be constant in each generation. Let 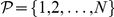. As mentioned above, shills are treated as normal agents by conforming to play rules. Meanwhile it is assumed that shills know nothing about normal agents' strategies, but they can remember and share the action sequence of normal agents playing with shills in the current generation. With this knowledge, they can estimate the level of cooperativity of normal agents and take suitable action. The simplest way of estimating a normal agent's strategy based on the action sequence is to calculate the frequency of cooperation. And then a shill uses it to decide appropriate reaction: to cooperate if the normal agent has high frequency of cooperation, otherwise to defect. This is what we called Frequency-based Tit for Tat (F-TFT). Note that F-TFT is a different form of strategy from normal agents' reactive strategy 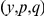. But this is allowed in soft control because shills can use their own strategies as long as they conform to play rules in the original group. We utilize F-TFT as a shill's strategy in the following part.

In each generation, all shills share knowledge 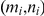 for normal agent 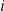 (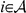), where 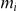 is how many stages agent 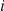 has interacted with shills so far and 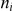 is the number of cooperation in 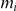 stages. At the beginning of each generation 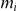 and 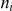 are initialized as 0. Then a shill with F-TFT uses 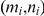 to make decisions: if 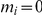, the shill cooperates; otherwise it cooperates with the probability 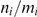. After a stage, 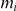 is increased by 1, and 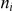 is increased by 1 if agent 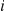 cooperates at that stage. Because each shill can access 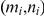, F-TFT is always based on the history of shills interacting with agent 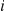 so far. Therefore at the end of each generation, 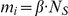 and 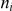 is the total number of cooperation that agent 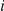 takes while playing with shills.

For any 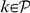, player 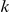
's total payoff 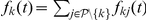 where 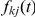 is the total payoff player 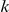 receives from interacting with player 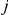. Rewrite Eq. (1) as below:
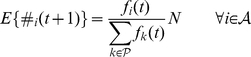
(2)


According to Eq. (2), note that the role of shills is to intervene in a normal agent's payoff through interacting with it. In fact, shills playing F-TFT reward cooperative behaviors and punish defective ones so as to promote cooperation.

## Results

The performance of soft control is studied through a series of numerical experiments. Simulation results presented in figures are averaged on 100 independent random realizations where 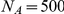 and a normal agent's strategy is uniformly generated in 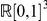. Besides, we assume that any player can be influenced by noise to take the opposite action with the probability 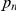 in each stage. In experiments let 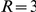, 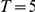, 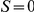 and 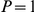 [1]. But our analytical proof (see in Appendix S1) illustrates the effectiveness of soft control under complete interaction for arbitrary 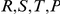 which satisfy 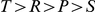 and 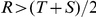.

### Survival of the fittest

Actually Eq. (2) reflects the idea of “survival of the fittest”, i.e. the more payoff one player gets, the more offspring it reproduces. Because shills are assumed to pose as normal agents, we first study the case that shills are also subject to “survival of the fittest”. In this scenario, we define the frequency of cooperation 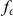 as the fraction of cooperation taken by players (i.e. normal agents and shills) in all games of one generation.

**Figure 1:**
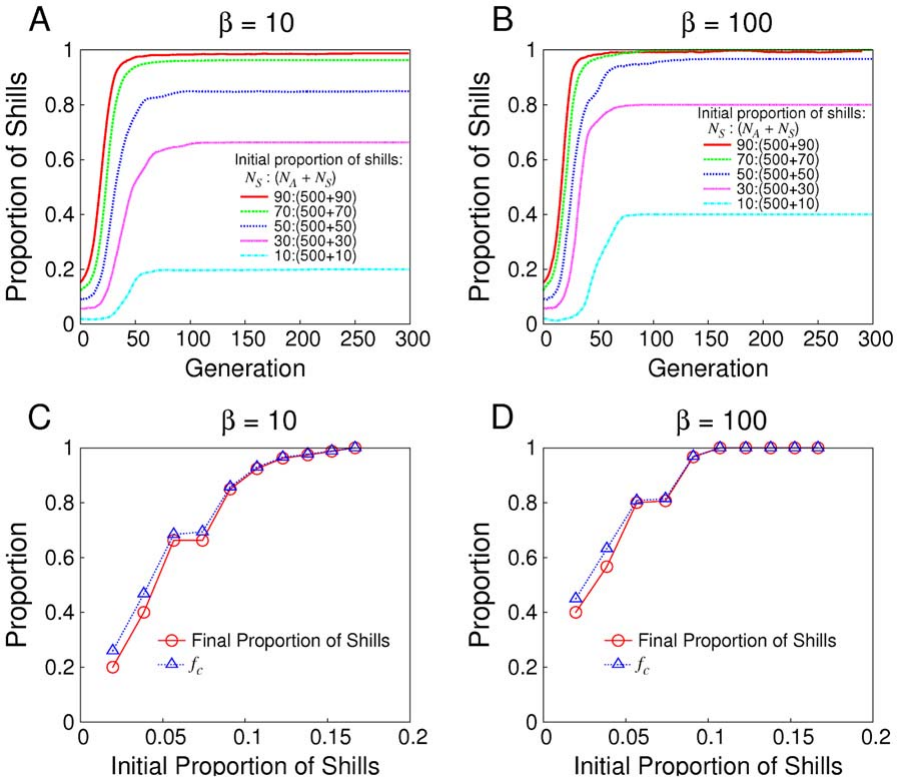
Shills are subject to survival of the fittest. (A) & (B) how the proportion of shills changes with different initializations when 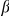 is 10 and 100 respectively. (C) & (D) the relationship between the proportion of shills and 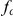 on 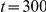 with different initializations when 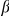 is 10 and 100 respectively.

The simulation results (Fig. 1) demonstrate that no matter in the short-term (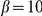) or long-term (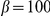) RPD, even though there is a small proportion (not less than 5% in the figure) of shills in the population, they will become the majority at last. Thus 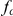 mainly derives from shills' action. So the cooperation level can be high since shills like to cooperate when the opponent cooperates. Soft control seems effective in this sense. But it is mainly due to the fact that shills win the game of “survival of the fittest” and replace normal agents. This is not so fair since shills get more information than normal agents. So we restrict the number of shills 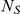 to be constant in following parts of simulations to see how soft control works. Therefore, 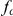 is defined as the fraction of cooperation taken by normal agents in all games of one generation.

### Evolution of 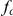 and strategies

**Figure 2:**
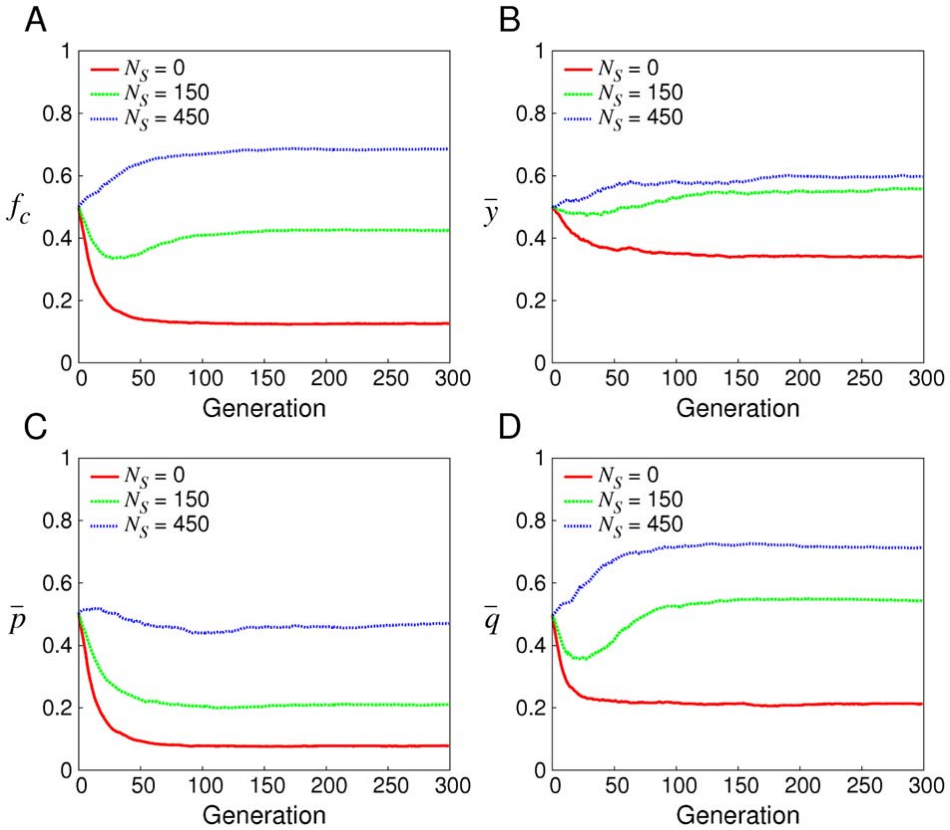
The evolution of 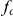 and strategies with different 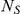. When 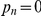 and 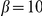, the evolution of 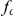, 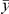, 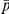 and 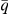 are demonstrated as 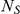 varies, where 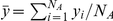, 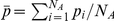 and 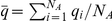.

Fig. 2 demonstrates the performance of soft control with various 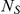. When 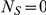, normal agents with smaller 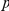 and 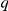 (i.e. less likely to cooperate when the opponent defects or cooperates in the last move respectively) get more payoff, which leads to the prevalence of defection. When defection prevails, 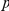 is more important than 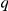 on determining a normal agent's payoff. So the red line in Fig. 2 (A) fits to the red line in Fig. 2 (C) well. Comparatively when 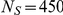, there are sufficient shills to make normal agents with larger 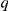 get more payoff by cooperating with them. Thus cooperation is beneficial such that cooperation dominates defection. Interestingly note that when 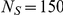, 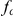 has a first decrease and then increases. The reason is that although cooperation is sustained by shills all the time, in the first period the number of shills is not large enough to ensure cooperation more profitable, which leads to the dominance of defection. But later, defection is no longer advantageous. On one hand defection is not supported by shills; on the other hand, playing defection only receives 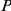 points rather than 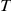 points in most interaction due to the prevalence of defection. But by contrast cooperation is more beneficial because it is supported by shills. Consequently 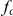 increases after the first period.

Above results indicate that after adding shills, cooperation is promoted. In the following part, we study soft control under other settings, which include the short-term (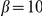) vs. long-term (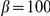) RPD, noise-free (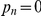) vs. noisy (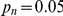) interaction, and sharing vs. non-sharing knowledge.

### Different settings

**Figure 3:**
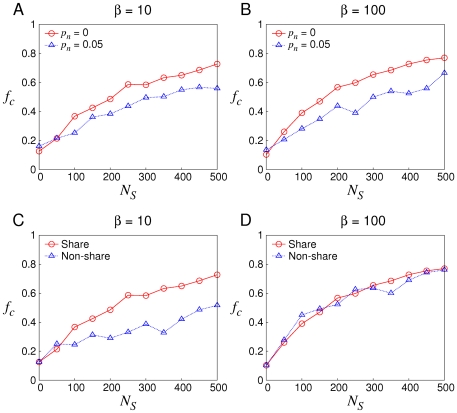
The performance of soft control under different settings. 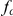
 on 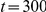 is shown because mostly after 100th generation, 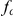 is convergent. (A) & (B) the sensitivity of the mechanism to noise when 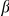 is 10 and 100 respectively. (C) & (D) the importance of sharing knowledge when 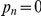, 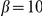 and 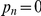, 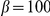 respectively.

Simulation results (Fig. 3 (A) & (B)) illustrate the robustness of the mechanism to noise. We find that soft control is slightly sensitive to noise. It is because the strategy F-TFT is on a basis of shared knowledge but noise causes shills' knowledge to be inaccurate. Also shills' own action is subject to noise. But mixed reactive strategies contain randomness, so noise in the interaction does not have a significant impact on the performance. In the meantime, we find that soft control is still efficacious to promote cooperation no matter in the short-term or long-term RPD. At this point, soft control is robust.

In order to evaluate the importance of knowledge on soft control, we compare the difference between sharing knowledge and non-sharing knowledge among shills for both the short-term and long-term RPD (Fig. 3 (C) & (D)). For the short-term RPD, sharing knowledge is better. Otherwise a shill does not have enough knowledge to estimate accurately the cooperativity of normal agents. In this situation, shills need to help each other, so sharing knowledge is crucial. However for the long-term RPD, this difference is no longer evident. It is because 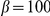 is sufficient for a shill to estimate its opponents even without knowledge providing from other shills. Thus sharing knowledge is not essential in this case. As a whole, sharing knowledge is rudimentary for the short-term RPD while it becomes dispensable for the long-term RPD.

Additionally, note that there is an inversely proportional relationship between 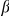 and 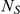. That is, to attain a given 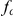, the required 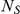 decreases as 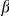 grows. The reason is that, for smaller 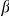 there have to be more shills to accumulate enough knowledge to estimate a normal agent accurately. Therefore as long as 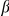 is sufficiently large, theoretically one shill can promote cooperation of the group.

### Incomplete population interaction

Above discussions are made in the complete population interaction case. But in real world systems it is not always like that. We should also consider how soft control works in the case of incomplete interaction, that is, players can interact with a proportion of the population. This proportion is denoted by 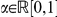 called the interaction locality (in the case of complete interaction, the proportion 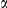 is equal to 1). In one generation, player 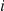 (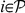) is chosen at random and then it randomly selects another one from 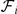 to play the 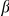-stage RPD once, where 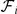 denotes as the set of players that player 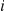 has never interacted with in the current generation. For normal agents, because they have no knowledge of others, their selection is random. But for shills, they can share knowledge and make full use of it. In this case, each shill 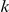 (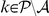) keeps its own knowledge 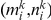 for normal agent 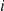 where 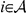. Shill 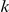 prefers to choose normal agents whose cooperative level (judged by 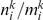, according to its knowledge) is higher than a threshold, 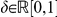, called the selection level. The set of these “qualified normal agents” is denoted as 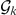. Shill 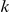 randomly selects a normal agent from 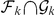 if not empty; otherwise it chooses from 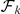 at random. After interacting with a normal agent, shill 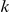 shares its knowledge with a proportion of other randomly chosen shills. This proportion is called the share proportion, denoted as 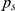. Above selection and interaction processes repeat until on average each player interacts with 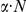 players. Then they reproduce offspring based on Eq. (2).

**Figure 4:**
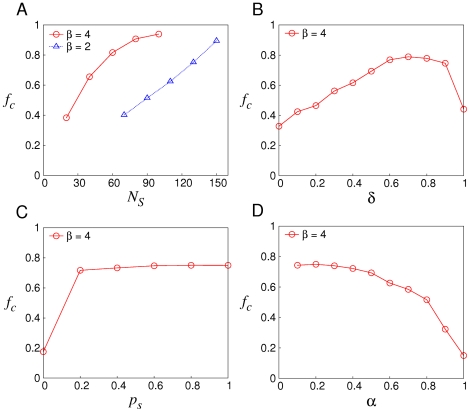
Effectiveness of soft control and its parametric sensitivity under incomplete population interaction. In this case if there is no extra declaration, parameters are 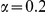, 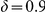, 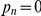 and 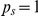. 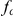 on 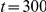 is demonstrated. (A) the relationship between 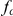 and 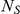 when 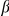 is 2 and 4 respectively. (B)(C)(D) the sensitivity to 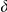, 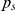 and 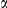 where 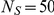 and 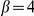.

Fig. 4 (A) illustrates the efficacy of soft control under incomplete interaction. And it also demonstrates an inversely proportional relationship between 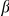 and 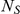. Note that compared to the case of complete interaction, cooperation is sustained for much smaller 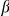 and 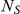 because shills' knowledge are used to choose opponents from normal agents as well. This dramatically enhances the performance of soft control.

In addition, the parametric sensitivity of soft control is studied for different values of 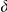, 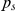 and 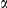. Fig. 4 (B) shows that for 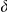, there is a tradeoff in the selection scheme of shills, which is similar to the relationship between exploration and exploitation. When 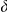 is large, there are few normal agents getting benefits from shills; whereas when 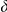 is small, shills' selection is almost random such that cooperative behaviors cannot get more rewards than defective ones. As a result playing cooperation is not advantageous. We also find that even though a shill shares its knowledge with a small proportion of other shills, soft control is still effective. Thus shills do not need to share with all others to promote cooperation (Fig. 4 (C)). It is noted that in Fig. 4 (D) as 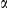 increases, incomplete interaction degenerates into complete interaction gradually such that shills lose the advantage on selection. Thus for a given 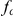, the required 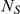 is proportional to 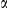.

### Mutation

**Figure 5:**
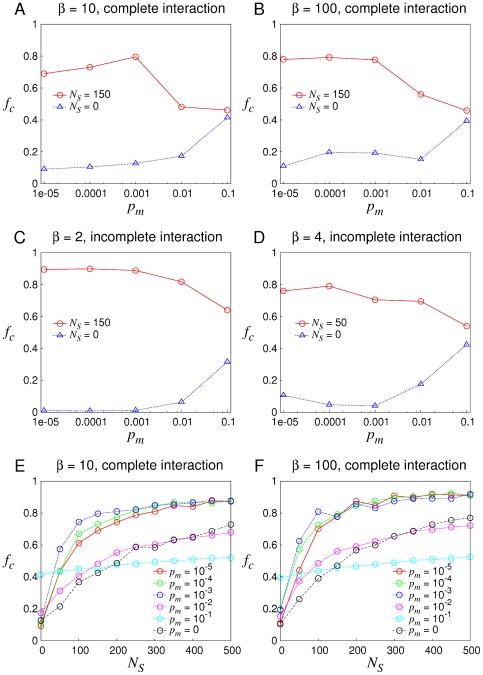
The effect of mutation in the reproduction. (A)–(D), the efficacy of soft control is demonstrated for 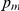 from 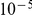 to 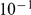, where (A) & (B) are under complete interaction with 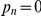 and (C) & (D) are under incomplete interaction with the parameters 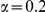, 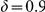, 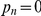 and 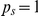. (E) & (F) under complete interaction, 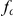 varies with the increase of 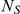 for different scales of 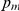.

We know that randomness derives not only from the strategy *per se* and noise in the interaction, but sometimes from strategy reproduction. So in the case of incomplete interaction, we investigate how soft control performs if randomness exists in strategy reproduction. Here 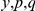 in a normal agent's strategy are represented as 10-bit binary string apiece. During reproduction, each bit in the string mutates from 0 to 1 or from 1 to 0 with the probability 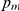 which is called the mutation probability. In Fig. 5 (A)–(D), we find that soft control can still promote cooperation when the order of magnitude of 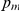 is no larger than 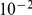. Meanwhile note that with the increase of 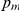, the capability of the mechanism becomes worse. This is due to the fact that for larger 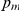, offspring are more different from their predecessor. As a result, any possible equilibrium becomes unstable any longer. Hence as long as 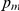 is not very large, cooperation is always promoted by adding shills. In Fig. 5 (E) & (F) it can be found that rare mutation (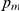 is not larger than 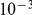) in reproduction is beneficial to increase the capability of soft control. In fact, small 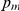 can increase the diversity of the strategy space such that there would be a possibility to incorporate higher cooperativity of normal agents while it does not destroy the established equilibrium.

## Discussion

In this paper we propose a mechanism called soft control to promote cooperation. For a group of agents playing the finitely but end-unknown RPD, the self-organized evolution of the population does not favor cooperation. However simulation results show that cooperation is promoted after introducing shills without violating play rules in the original group. Meanwhile the performance of soft control is studied under different settings, which include short-term vs. long-term RPD, noise-free vs. noisy, and complete vs. incomplete population interaction. We find that the mechanism is slightly sensitive to noise but still effective. At this point, soft control is robust to noise. In the short-term RPD, sharing knowledge is essential to shills while it becomes unimportant in the long-term RPD. Cooperation can be promoted by shills in both complete and incomplete interaction case. Yet with selection based on knowledge, it is more efficient for shills to promote cooperation in the incomplete interaction case than in the complete interaction case. We find that shills also perform well in both the complete and the incomplete interaction case even with mutation in strategy reproduction, and rare mutation is beneficial to increase the capability of soft control. Our results demonstrate that, to achieve a given cooperation level, the required number of shills is inversely proportional to the time period of games, but proportional to the interaction locality. In addition the effectiveness of soft control under complete interaction is proven analytically in the appendix.

There are several literatures relevant to the intervention in individual behaviors [60], [61]. In [60], authors investigate how a teacher guides a learner to cooperate in the Prisoner's Dilemma. The role of the teacher is similar to that of a shill, but they focus on the learning scheme in 2-agent games. Authors in [61] explore the effect of three different kinds of special agents, namely radicals, revolutionaries and reactionaries, on the transition of regimes. It has dramatically changed the transition time. Different from shills, those agents utilize strategies without feedback knowledge.

In our study it is required that shills should pose as normal agents by complying with play rules. This is the main point of soft control, to keep play rules in the original group unchanged. What is more, it has additional reasons in this paper: if shills are treated as special agents, a normal agent may behave differently on interacting with shills and other normal agents. It may pretend to be a cooperator in order to get benefits from shills, but act as a defector to exploit other normal agents. In this situation mutual defection is still the only consequence. Therefore we stress the importance of a shill being treated as a normal agent by truly normal ones.

This paper is the first step to study soft control in the well-mixed population, and more extensions deserve our further efforts to explore. It is interesting to study soft control based on other strategy sets besides reactive strategies, such as deterministic finite automata [56], [62], look-up table [1], Turing machines [63] and neural networks [64]. In our further study, we will also investigate whether F-TFT is the best strategy for shills and the properties of the best strategy for the specific scenario.

We will extend soft control to structured populations and study the influence of different spatial structures (e.g. the regular network, the random network and the scale-free network) on the mechanism. In fact, the network topology appears in many real-world systems. The models with the spatial structure display different properties (such as pattern formation and diffusion [31], [65]) from the mean-field type model. Consider soft control in the case of structured populations: except the number and the strategy of shills, we also need to decide which nodes (normal agents) these shills should link to and how many links there are for each shill. Different networks might need different linking schemes. We know that in many networks, some nodes (such as hubs, nodes with high centrality, etc.) have more impact than the others on the overall performance. So it is crucial for shills to select important nodes to affect. The linking scheme will influence the performance of soft control. Notice that the importance of a node is also related to the dynamics of the system. So there might not exist a general heuristics of node selection for all systems. But some common principles might be discovered. On the other hand, adding links will increase cost in some systems. The trade-off between the performance and the cost will be another important topic of soft control.

Soft control can be viewed as a way of intervention in collective behaviors. It does not focus on how to re-design play rules of every agent for the desired purpose, but on how to induce our desired collective behaviors without changing play rules. At this point, soft control provides a possible direction for the study of reciprocal behaviors and it may be applied to other scenarios like Public Goods Game and Fashion Game, and to hinder the spread of panic/rumor in crowd or to control dynamical behaviors of other systems. Additionally it is necessary to study the applicability and limitation of soft control. Inspired by control theory [66], we will define and analyze the controllability of soft control in a general framework, i.e. to search for conditions that soft control can lead the system to the expected behavior. We believe that the controllability will relate to the jointly connectivity (or alike) of the system, which indicates every normal agent should be affected by shills directly or indirectly. We also believe that there will be a critical value of shill numbers or impact strength (which varies in different systems) to achieve the soft-control goal. Research following this line will provide a deep insight to soft control.

## Supporting Information

Appendix S1 The proof of the effectiveness under complete population interaction.
(PDF)
