# Supplementary material for: Special Agents Can Promote Cooperation in the Population
Source: PLoS One. 2011 Dec 21;6(12):e29182. doi: 10.1371/journal.pone.0029182 (PMC3244459; doi:10.1371/journal.pone.0029182)
Supplement: Appendix S1 — The proof of the effectiveness under complete population interaction. (PDF) [file pone.0029182.s001.pdf]

Appendix S1: The proof of the effectiveness under complete population interaction

Xin Wang, Jing Han*, Huawei Han
Key Laboratory of Systems and Control, Institute of Systems Science, Academy of Mathematics and Systems Science, Chinese Academy of Sciences, Beijing 100190, China
∗ E-mail: Corresponding hanjing@amss.ac.cn

In the appendix, the following proposition of the effectiveness under complete interaction is proved:

Proposition. *Assume that the population plays the β-stage RPD ($\beta \geq 1$) for any given R, S, T, P which satisfy $T > R > P > S$ and $R > (T+S)/2$. The types of mixed reactive strategies n is sufficiently large to contain any possible strategy. Also assume that shills use the strategy F-TFT. Then $\exists x^* \geq 0$, when the proportion of shills is larger than x^*, the frequency of cooperation f_c converges to one.*

Before we prove, some preparations are necessary for the mathematical analysis.

As is known, the transformation $R^{'} = R - S, S^{'} = S - S, T^{'} = T - S, P^{'} = P - S$ does not alter the equilibrium point. So for any given R, S, T, P, firstly we make this transformation to have $S = 0$ in following proofs without declaring any more.

Assume that there are n types of mixed reactive strategies for normal agents. Let $\mathcal{A} = \{1, 2, \ldots, n\}$ and $\mathcal{P} = \mathcal{A} \cup \{n+1\} = \{1, 2, \ldots, n+1\}$. The ith type of strategy is denoted as s_i, $i \in \mathcal{A}$. We also denote as s_{n+1} shills' strategy F-TFT. Let $x_i(t)$, $i \in \mathcal{P}$, denote as the proportion of a player with s_i in t generation. They satisfy $\sum_{i\in\mathcal{P}} x_i(t) = 1$ for all $t \geq 0$. Denote as $f(s_i|s_j)$ the expected total payoff that a player with s_i receives from playing with a player with s_j for the β-stage RPD. Then the expected total payoff of the player with s_i is $f(s_i|\bar{s}(t)) = \sum_{j\in\mathcal{P}} f(s_i|s_j)x_j(t)$. We also denote as $f(\bar{s}(t)|\bar{s}(t)) = \sum_{i\in\mathcal{P}} x_i(t) \sum_{j\in\mathcal{P}} f(s_i|s_j)x_j(t)$ the average expected total payoff of the population. The reproduction rule is actually the discrete-time replicator dynamics, rewritten as follows for $i \in \mathcal{P}$:

$$x_i(t+1) = x_i(t)\frac{f(s_i|\bar{s}(t))}{f(\bar{s}(t)|\bar{s}(t))} \qquad \text{for } t \geq 0. \tag{1}$$

Then we define a set $E = \{s_i : x_i(\infty) = \lim_{t\to\infty} x_i(t) > 0\}$, which consists of those strategies existing at the end. Note that here the definition of the limit in E is general, i.e. the trajectory of $x_i(t)$ may approach to a limit cycle. It is easy to check that the set E is nonempty. Also denote the payoff of a player with s_i in E by $f(s_i|\bar{s}(\infty)) = \lim_{t\to\infty} f(s_i|\bar{s}(t))$. We give key properties of E in Lemma 1, which extends the lemma in [1]:

Lemma 1. *In the set E,*
(1) if $\exists t_0 > 0$, when $t > t_0$, $f(s_i|\bar{s}(t)) \geq f(s_j|\bar{s}(t))$, then if $s_j \in E$, we get $s_i \in E$;
(2) there do not exist the strategies s_i, s_j in E such that either $f(s_i|\bar{s}(\infty)) > f(s_j|\bar{s}(\infty))$ or $f(s_j|\bar{s}(\infty)) > f(s_i|\bar{s}(\infty))$ holds all the time.

Proof. If E contains only one element, two above arguments obviously hold. As follows we consider the case that there are at least two elements in E.

(1) We prove the contrapositive form of the first argument, i.e. if conditions are satisfied, $s_i \notin E$ implies that $s_j \notin E$. If $s_i \notin E$,, that is, $\lim_{t\to\infty} x_i(t) = 0$, then

$$0 < x_j(t) = x_j(0)\prod_{l=0}^{t-1}\frac{f(s_j|\bar{s}(l))}{f(\bar{s}(l)|\bar{s}(l))} \leq \frac{x_j(t_0+1)}{x_i(t_0+1)}x_i(t) \to 0 \tag{2}$$

So $\lim_{t\to\infty} x_j(t) = 0$, that is, $s_j \notin E$.

(2) Suppose that $f(s_i|\bar{s}(\infty)) > f(s_j|\bar{s}(\infty))$ holds all the time. When t is large enough, we get

$$\frac{x_i(t+1)}{x_j(t+1)} = \frac{f(s_i|\bar{s}(t))}{f(s_j|\bar{s}(t))} \cdot \frac{x_i(t)}{x_j(t)} \geq M \cdot \frac{x_i(t)}{x_j(t)} \tag{3}$$

where $M > 1$. So $\frac{x_i(t)}{x_j(t)}$ increases monotonically. If $\lim_{t\to\infty} \frac{x_i(t)}{x_j(t)} = \infty$, we get $\lim_{t\to\infty} x_j(t) = 0$ due to the boundedness of $x_i(t)$. It contradicts with $s_j \in E$. If $\lim_{t\to\infty} \frac{x_i(t)}{x_j(t)} < \infty$, we take the limit operation to both side of inequality (3) and get $\lim_{t\to\infty} \frac{x_i(t+1)}{x_j(t+1)} \geq M \cdot \lim_{t\to\infty} \frac{x_i(t)}{x_j(t)}$, which is impossible. Therefore the conclusion is proven. □

Lemma 2. *For any two number sequences $\{\alpha_n\}_{n\geq 1}$ and $\{\beta_{i,n}\}_{n\geq 1, i\leq n}$, their limit satisfy:*
(1) $\alpha_n \to a$ with $n \to \infty$;
(2) $\beta_{i,n} \to 0$ with $n \to \infty$, for given i;
(3) $\sum_{i=1}^n \beta_{i,n} \to b$ with $n \to \infty$ and $\sum_{i=1}^n |\beta_{i,n}|$ is bounded.
then

$$\lim_{n\to\infty} \sum_{i=1}^n \alpha_i \beta_{i,n} = a \cdot b \tag{4}$$

Proof. According to the definition of number sequence limit, it is known that
(1) $\forall \varepsilon_1 > 0$, $\exists N_1$, when $n > N_1$, $|\alpha_n - a| < \varepsilon_1$;
(2) For given i, $\forall \varepsilon_{2,i} > 0$, $\exists N_{2,i}$, when $n > N_{2,i}$, $|\beta_{i,n}| < \varepsilon_{2,i}$;
(3) $\forall \varepsilon_3 > 0$, $\exists N_3$, when $n > N_3$, $|\sum_{i=1}^n \beta_{i,n} - b| < \varepsilon_3$.
Thereby

$$\left|\sum_{i=1}^n \alpha_i \beta_{i,n} - ab\right| \leq \left|\sum_{i=1}^n (\alpha_i - a)\beta_{i,n}\right| + |a| \cdot \left|\sum_{i=1}^n \beta_{i,n} - b\right| \tag{5}$$

For the rightmost item of (5), $|a| \cdot |\sum_{i=1}^n \beta_{i,n} - b| < |a| \cdot \varepsilon_3$ when $n > N_3$. In the meantime,

$$\left|\sum_{i=1}^n (\alpha_i - a)\beta_{i,n}\right| \leq \left|\sum_{i=1}^N (\alpha_i - a)\beta_{i,n}\right| + \left|\sum_{i=N+1}^n (\alpha_i - a)\beta_{i,n}\right| \tag{6}$$

where we take $N \geq N_1$, which implies $n > N_1$. Then

$$\left|\sum_{i=N+1}^n (\alpha_i - a)\beta_{i,n}\right| \leq \sum_{i=N+1}^n |\alpha_i - a| \cdot |\beta_{i,n}| < \varepsilon_1 \cdot H \tag{7}$$

where H is the bound of $\sum_{i=1}^n |\beta_{i,n}|$. When $n > N_2 = \max\{N_{2,i}\}$ where $i = 1, 2, \ldots, N$,

$$\left|\sum_{i=1}^N (\alpha_i - a)\beta_{i,n}\right| \leq \sum_{i=1}^N |\alpha_i - a| \cdot |\beta_{i,n}| < \varepsilon_2 \cdot \sum_{i=1}^N |\alpha_i - a| \tag{8}$$

where $\varepsilon_2 = \max\{\varepsilon_{2,i}\}$ for $i = 1, 2, \ldots, N$. Take $N^* = \max\{N_j\}$ and $\varepsilon > 3 \cdot \max\{\varepsilon_j \cdot M_j\}$ for $j = 1, 2, 3$, where $M_1 = H$, $M_2 = \sum_{i=1}^N |\alpha_i - a|$ and $M_3 = |a|$, when $n > N^*$, $|\sum_{i=1}^n \alpha_i \beta_{i,n} - ab| < \varepsilon/3 + \varepsilon/3 + \varepsilon/3 = \varepsilon$, i.e., $\sum_{i=1}^n \alpha_i \beta_{i,n} \to a \cdot b$ as $n \to \infty$. □

Lemma 3. *Assume that the population plays the β-stage RPD. For a normal agent with the strategy $(y,p,q)\in\mathbb{R}[0,1]^3$, the probability that shills with F-TFT cooperate is convergent to $\frac{y+(\beta-1)p}{\beta-(\beta-1)\lambda}$ as sharing times tend to be infinite, where $\lambda=q-p$.*

Proof. Let $b(\gamma,m)$ denote as the probability that a shill cooperates after m-th stage ($m=1,2,\ldots,\beta$) when knowledge of the normal agent is shared for γ times. As follows we prove that for $m=1,2,\ldots,\beta$, $b(\gamma,m)\to\frac{y+(\beta-1)p}{\beta-(\beta-1)\lambda}$ as $\gamma\to\infty$. At first consider the limit of $b(\gamma,\beta)$ as $\gamma\to\infty$. According to the update scheme, it can be calculated that

$$b(\gamma,m)=\begin{cases}\frac{y+\beta\gamma\cdot b(\gamma-1,\beta)}{\beta\gamma+1} & m=1\\ \frac{(\beta\gamma+m-1+\lambda)\cdot b(\gamma,m-1)+p}{\beta\gamma+m}+\frac{\lambda\triangle b(\gamma,m-1)}{\beta\gamma+m} & m=2,3,\ldots,\beta\end{cases} \tag{9}$$

where $b(\gamma,0)=b(\gamma-1,\beta)$ and $\triangle b(\gamma,m-1)=b(\gamma,m-2)-b(\gamma,m-1)$, $m=2,3,\ldots,\beta$. So

$$\begin{aligned} b(\gamma,\beta) &= f(\gamma)b(\gamma-1,\beta)+g(\gamma)y+h(\gamma)p+\lambda\triangle E(\gamma)\\ &\cdots\\ &= b(0,\beta)\prod_{i=1}^{\gamma}f(i)+y\sum_{i=1}^{\gamma}g(i)\prod_{j=i+1}^{\gamma}f(j)+\\ &\quad p\sum_{i=1}^{\gamma}h(i)\prod_{j=i+1}^{\gamma}f(j)+\lambda\sum_{i=1}^{\gamma}\triangle E(i)\prod_{j=i+1}^{\gamma}f(j)\end{aligned} \tag{10}$$

where

$$c(i,j) = 1+\frac{\lambda}{\beta i+j} \tag{11}$$

$$f(i) = \frac{i}{i+1}\prod_{j=1}^{\beta-1}c(i,j) \tag{12}$$

$$g(i) = \frac{1}{\beta(i+1)}\prod_{j=1}^{\beta-1}c(i,j) \tag{13}$$

$$h(i) = \frac{1}{\beta(i+1)}\sum_{j=1}^{\beta-1}\prod_{k=j+1}^{\beta-1}c(i,k) \tag{14}$$

$$\triangle E(i) = \frac{1}{\beta(i+1)}\sum_{j=1}^{\beta-1}\triangle b(i,j)\prod_{k=j+1}^{\beta-1}c(i,k) \tag{15}$$

In the following we calculate the limit of $b(\gamma,\beta)$ in (10) as $\gamma\to\infty$.

First for $\lim_{\gamma\to\infty}\prod_{i=1}^{\gamma}f(i)$. Due to $|\lambda|\le 1$,

$$0<f(i)\le\frac{\beta i}{\beta i+1}=1-\frac{1}{\beta i+1}<e^{-\frac{1}{\beta i+1}} \tag{16}$$

and

$$\lim_{\gamma\to\infty}\prod_{i=1}^{\gamma}e^{-\frac{1}{\beta i+1}}=\lim_{\gamma\to\infty}e^{-O(\log\gamma)}=0 \tag{17}$$

So $\lim_{\gamma\to\infty}\prod_{i=1}^{\gamma}f(i)=0$.

Then for $\lim_{\gamma\to\infty}\sum_{i=1}^{\gamma} g(i)\prod_{j=i+1}^{\gamma} f(j)$. Let $G(\gamma)=\prod_{i=1}^{\beta-1} c(\gamma,i)$, thus

$$\sum_{i=1}^{\gamma} g(i)\prod_{j=i+1}^{\gamma} f(j) = \frac{1}{\beta(\gamma+1)}\sum_{i=0}^{\gamma-1}\prod_{j=0}^{i} G(\gamma-j) \tag{18}$$

$$= \frac{F(\gamma)}{\beta(\gamma+1)} \tag{19}$$

where $F(\gamma)=\sum_{i=0}^{\gamma-1}\prod_{j=0}^{i} G(\gamma-j)$. For $\gamma\geq 1$, it holds that $\left(1+\frac{\lambda}{\beta(\gamma+1)}\right)^{\beta-1}\leq G(\gamma)\leq\left(1+\frac{\lambda}{\beta\gamma}\right)^{\beta-1}$, so we obtain $F(\gamma)$'s upper bound $\overline{F}(\gamma)$ and lower bound $\underline{F}(\gamma)$. Expand $\overline{F}(\gamma)$ as λ's polynomial and we can calculate that $\lim_{\gamma\to\infty}\frac{\overline{F}(\gamma)}{\beta(\gamma+1)}=\frac{1}{\beta-(\beta-1)\lambda}$. Similarly $\lim_{\gamma\to\infty}\frac{\underline{F}(\gamma)}{\beta(\gamma+1)}=\frac{1}{\beta-(\beta-1)\lambda}$. So $\lim_{\gamma\to\infty}\frac{F(\gamma)}{\beta(\gamma+1)}=\frac{1}{\beta-(\beta-1)\lambda}$, that is, $\lim_{\gamma\to\infty}\sum_{i=1}^{\gamma} g(i)\prod_{j=i+1}^{\gamma} f(j)=\frac{1}{\beta-(\beta-1)\lambda}$.

Third for $\lim_{\gamma\to\infty}\sum_{i=1}^{\gamma} h(i)\prod_{j=i+1}^{\gamma} f(j)$. It can be checked that $\lim_{\gamma\to\infty}\frac{h(\gamma)}{g(\gamma)}=\beta-1$. Then

$$\lim_{\gamma\to\infty}\sum_{i=1}^{\gamma} h(i)\prod_{j=i+1}^{\gamma} f(j)=\lim_{\gamma\to\infty}\sum_{i=1}^{\gamma}\frac{h(i)}{g(i)}\cdot g(i)\prod_{j=i+1}^{\gamma} f(j) \tag{20}$$

According to Lemma 2, we calculate that $\lim_{\gamma\to\infty}\sum_{i=1}^{\gamma} h(i)\prod_{j=i+1}^{\gamma} f(j)=\frac{\beta-1}{\beta-(\beta-1)\lambda}$.

Finally for $\lim_{\gamma\to\infty}\sum_{i=1}^{\gamma}\triangle E(i)\prod_{j=i+1}^{\gamma} f(j)$. Here we testify $\lim_{\gamma\to\infty}\frac{\triangle E(\gamma)}{h(\gamma)}$. Note that for $1\leq m\leq\beta-1$, $\lim_{\gamma\to\infty}\triangle b(\gamma,m)=0$. Then $\lim_{\gamma\to\infty}\frac{\triangle E(\gamma)}{h(\gamma)}=\lim_{\gamma\to\infty}\frac{\sum_{i=1}^{\beta-1}\triangle b(\gamma,i)\prod_{j=i+1}^{\beta-1}c(j,\gamma)}{\sum_{i=1}^{\beta-1}\prod_{j=i+1}^{\beta-1}c(j,\gamma)}=0$. So according to Lemma 2, $\lim_{\gamma\to\infty}\sum_{i=1}^{\gamma}\triangle E(i)\prod_{j=i+1}^{\gamma} f(j)=0$.

In sum, $\lim_{\gamma\to\infty} b(\gamma,\beta)=\frac{y+(\beta-1)p}{\beta-(\beta-1)\lambda}$. According to (9), we can calculate that for $m=1,2,\ldots,\beta$, $\lim_{\gamma\to\infty} b(\gamma,m)=\frac{y+(\beta-1)p}{\beta-(\beta-1)\lambda}$. □

Next we derive our proof of the effectiveness of soft control under complete interaction. As is illustrated in Fig. 1, shills can replace normal agents. For the purpose of studying soft control to promote cooperation in normal agents, we restrict the proportion of shills to be constant in each generation.

Proposition. *Assume that the population plays the β-stage RPD ($\beta\geq 1$) for any given R,S,T,P which satisfy $T>R>P>S$ and $R>(T+S)/2$. The types of mixed reactive strategies n is sufficiently large to contain any possible strategy. Also assume that shills use the strategy F-TFT. Then $\exists x^*\geq 0$, when the proportion of shills is larger than x^*, the frequency of cooperation f_c converges to one.*

Proof. According to Lemma 3, for a normal agent with the strategy $s=(y,p,q)$, a shill cooperates with the probability $\frac{y+(\beta-1)p}{\beta-(\beta-1)\lambda}$ approximately after finite interactions.

The payoff of an s agent can be expressed as

$$f(s|\bar{s}(t))=\sum_{i\in\mathcal{A}} f(s|s_i)x_i(t)+x_{n+1}\cdot f(s|s_{n+1}) \tag{21}$$

Let $f(s|\hat{s}(t))=\sum_{i\in\mathcal{A}} f(s|s_i)x_i(t)$ and $\tilde{y}=\frac{y+(\beta-1)p}{\beta-(\beta-1)\lambda}$, then $f(s|s_{n+1})$ can be expanded as

$$f(s|s_{n+1}) = \begin{pmatrix} y & 1-y\end{pmatrix}\begin{pmatrix} R & 0\\ T & P\end{pmatrix}\begin{pmatrix}\tilde{y}\\ 1-\tilde{y}\end{pmatrix} \tag{22}$$

$$+(\beta-1)\cdot\begin{pmatrix}\tilde{y} & 1-\tilde{y}\end{pmatrix}\begin{pmatrix} q & 1-q\\ p & 1-p\end{pmatrix}\begin{pmatrix} R & 0\\ T & P\end{pmatrix}\begin{pmatrix}\tilde{y}\\ 1-\tilde{y}\end{pmatrix} \tag{23}$$

$$= \beta(P+(T-2P)\tilde{y}+(R+P-T)\tilde{y}^2) \tag{24}$$

where $0 \leq \tilde{y} \leq 1$. For above expression, three cases are considered:

(1) When $R + P = T$, we get $T > 2P$, so the maximum of $f(s|s_{n+1})$ is reached at $\tilde{y} = 1$;

(2) When $R + P < T$, the symmetry axis of $f(s|s_{n+1})$ relative to $\tilde{y}$ is $\frac{T-2P}{2(T-R-P)} > 1$ because $2R > T$ which is obtained by one of restrictions to the payoff matrix $R > \frac{T+S}{2}$ when $S = 0$. Hence the maximum of $f(s|s_{n+1})$ is reached at $\tilde{y} = 1$;

(3) When $R+P > T$, the symmetry axis of $f(s|s_{n+1})$ relative to $\tilde{y}$ is $\frac{T-2P}{2(T-R-P)} = \frac{1}{2}\left(1 + \frac{R-P}{T-R-P}\right) < \frac{1}{2}$, thus the maximum of $f(s|s_{n+1})$ is also reached at $\tilde{y} = 1$.

So for arbitrary R, S, T, P satisfying conditions, the maximum of $f(s|s_{n+1})$ is reached at $\tilde{y} = 1$, i.e. $y = 1$, $q = 1$. Take $s^*(t) = \arg\max_{s=(1,p,1)} f(s|\hat{s}(t))$. Let $x^*(t) = \max\{0, \max_{y_i,q_i \neq 1} \frac{f(s_i|\hat{s}(t)) - f(s^*(t)|\hat{s}(t))}{f(s^*(t)|s_{n+1}) - f(s_i|s_{n+1})}\} \geq 0$. Then when $x_{n+1} > x^* = \sup_{t \geq 0} x^*(t)$, we get $\forall t \geq 0, f(s^*(t)|\bar{s}(t)) > f(s_i|\bar{s}(t))$ for all $s_i \neq s^*(t)$, so there exists the strategy with $y = 1$ and $q = 1$ in E. And based on Lemma 1, we can prove that E contains such the strategy exclusively. Therefore f_c converges to one. □

Note that the above proposition also holds when we consider the payoff matrix $T = b > R = 1 > P = S = 0$ where $b < 2$ [2].

References

1. Nachbar JH (1990) Evolutionary selection dynamics in games: convergence and limit properties. International Journal of Game Theory 19: 59–89.

2. Nowak M, May R (1992) Evolutionary games and spatial chaos. Nature 359: 826–829.
